# Identifying novel potential drug targets for endometriosis via plasma proteome screening

**DOI:** 10.3389/fendo.2024.1416978

**Published:** 2024-07-05

**Authors:** Tian Tao, Xiaoyu Mo, Liangbin Zhao

**Affiliations:** ^1^ Department of Nephrology, Hospital of Chengdu University of Traditional Chinese Medicine, Chengdu, China; ^2^ Department of Gynaecology and Obstetrics, West China Second Hospital, Sichuan University, Chengdu, China

**Keywords:** endometriosis, drug target, plasma proteome, Mendelian randomization, proteome-wide association study

## Abstract

**Background:**

Endometriosis (EM) is a chronic painful condition that predominantly affects women of reproductive age. Currently, surgery or medication can only provide limited symptom relief. This study used a comprehensive genetic analytical approach to explore potential drug targets for EM in the plasma proteome.

**Methods:**

In this study, 2,923 plasma proteins were selected as exposure and EM as outcome for two-sample Mendelian randomization (MR) analyses. The plasma proteomic data were derived from the UK Biobank Pharmaceutical Proteomics Project (UKB-PPP), while the EM dataset from the FinnGen consortium R10 release data. Several sensitivity analyses were performed, including summary-data-based MR (SMR) analyses, heterogeneity in dependent instruments (HEIDI) test, reverse MR analyses, steiger detection test, and bayesian co-localization analyses. Furthermore, proteome-wide association study (PWAS) and single-cell transcriptomic analyses were also conducted to validate the findings.

**Results:**

Six significant (p < 3.06 × 10^-5^) plasma protein-EM pairs were identified by MR analyses. These included EPHB4 (OR = 1.40, 95% CI: 1.20 - 1.63), FSHB (OR = 3.91, 95% CI: 3.13 - 4.87), RSPO3 (OR = 1.60, 95% CI: 1.38 - 1.86), SEZ6L2 (OR = 1.44, 95% CI: 1.23 - 1.68) and WASHC3 (OR = 2.00, 95% CI: 1.54 - 2.59) were identified as risk factors, whereas KDR (OR = 0.80, 95% CI: 0.75 - 0.90) was found to be a protective factor. All six plasma proteins passed the SMR test (P < 8.33 × 10^-3^), but only four plasma proteins passed the HEIDI heterogeneity test (PHEIDI > 0.05), namely FSHB, RSPO3, SEZ6L2 and EPHB4. These four proteins showed strong evidence of co-localization (PPH4 > 0.7). In particular, RSPO3 and EPHB4 were replicated in the validated PWAS. Single-cell analyses revealed high expression of SEZ6L2 and EPHB4 in stromal and epithelial cells within EM lesions, while RSPO3 exhibited elevated expression in stromal cells and fibroblasts.

**Conclusion:**

Our study identified FSHB, RSPO3, SEZ6L2, and EPHB4 as potential drug targets for EM and highlighted the critical role of stromal and epithelial cells in disease development. These findings provide new insights into the diagnosis and treatment of EM.

## Introduction

Endometriosis (EM) is a condition in which endometrial glands and mesenchyme grow outside the uterine cavity (1). EM currently affects almost 10% of women of reproductive age worldwide (2, 3). Progressive chronic pain caused by recurrent bleeding from ectopic endometrial tissues can severely disrupt the patient’s life and work. Chronic pelvic inflammation and adhesions can also lead to infertility in reproductive women (4, 5). At present, there are no biomarkers available to diagnose EM. The gold standard for diagnosing EM is laparoscopic pathological biopsy (6). Furthermore, there is currently no treatment available to entirely cure EM. Recurrence is common following surgical resection, and existing medications for EM provide only limited symptom relief (7). Therefore, there is an urgent need to discover novel diagnostic biomarkers and drug targets for EM.

More and more drugs are being approved on the basis of genetic evidence. It is reported that up to 66% (33/50) of new drugs approved in 2021 alone are supported by genetics (8). With the vigorous development of high-throughput technology, thousands of protein quantitative trait loci (PQTL) have been identified, providing valuable data resources for biomarker exploration and drug discovery (9, 10). Mendelian randomization (MR) is an essential method for assessing genetic causality in epidemiological research. Single nucleotide polymorphisms (SNPs) associated with pQTLs have been selected as instrumental variables (IVs) for MR analyses to infer the direct causality between protein levels and disease. This approach is presently employed to identify novel disease biomarkers and drug targets for conditions like cancer, coronary heart disease, and autoimmune disorders (11–14). Nevertheless, its application in EM remains unexplored.

This study employs a comprehensive genetic analytical approach to explore potential drug targets for EM in the plasma proteome. First, based on recently published data from genome-wide association studies (GWAS) of the proteome, we selected the top SNPs in the cis region of plasma proteins as IVs for MR analyses to infer the possible causality between the circulating proteome and EM. Then, summary-data-based MR (SMR) analyses, heterogeneity in dependent instruments (HEIDI) test, reverse MR analyses, steiger detection test, and bayesian co-localization analyses were used for sensitivity analyses. Finally, proteome-wide association study (PWAS) and single-cell transcriptomic analyses were also performed to validate the findings.

## Methods

### Data sources of PQTL

Data for the pQTLs in the MR study were obtained from the UK Biobank Pharmaceutical Proteomics Project (UKB-PPP) (10). This project is a large-scale proteomic characterization study of 54,219 UK Biobank participants, and we selected 2,923 pQTLs from the baseline cohort (n = 34,557) (https://www.synapse.org/Synapse:syn51365301) for analyses. This is because the baseline cohort provides a good representation of the overall level of UKB participants in terms of gender, ethnic background, and blood type. In addition, we used plasma proteins data from the Atherosclerosis Risk in Communities (ARIC) study (15), for a validated PWAS study to increase the credibility of the MR analyses. The ARIC study is also a large-scale proteomic project that includes genetic data on 4,657 plasma proteins from 7,213 European Americans and 1,871 African Americans. In this study, we used 2004 proteomic data from 7,213 European Americans from this project.

### Data sources of endometriosis

The FinnGen GWAS database contains extensive genotypic and phenotypic data from Finnish national health registries and is utilized by researchers worldwide (16). The EM dataset for MR analyses was derived from the R10 release data of the FinnGen database, with a total of 128,171 participants of European ancestry, including 16,588 cases and 111,583 controls (https://r10.finngen.fi/pheno/N14_ENDOMETRIOSIS). The diagnostic criteria for EM for MR and PWAS analyses is the “N80” in ICD-10.

### Preliminary MR analyses

In this study, the SNPs within the cis regions (within 1 Mb of the transcription start site of each protein-coding gene) of pQTLs that met the criteria of P<5x10^-8^ and r^2^<0.1 were defined as the top SNPs of cis-pQTLs. These top SNPs of cis-pQTLs were then used as IVs to represent plasma proteins (17). The causality between plasma proteins and EM was then investigated in a two-sample MR study. For plasma proteins with only one top SNP, the Wald ratio method was used to assess the causality between them and EM, and for plasma proteins with multiple top SNPs, the stronger statistical power of the Inverse variance weighting (IVW) method was used (18). This part of the work was performed using the “TwoSampleMR” package of the R software (19). F-values were used to assess the strength of association of each genetic variant with exposure. Generally, F > 10 indicates the absence of weak IVs. The F value was calculated using the formula F = R^2^ * (N - 1 - K)/(1 - R^2^) * K, where R^2^ = 2*(1-MAF)*MAF*β/SD (MAF is the minor allele frequency, β is the effect value of the allele and SD is the standard error of β. N is the number of exposed samples and K is the number of IVs). The MAF of all SNPs was set to > 0.01 to avoid the effect of rare genetic variants (**Supplementary Table S1**). Cochran’s Q test and horizontal pliotropy test were employed to assess the sensitivity of the IVs (20, 21). The delta method was used to estimate the corresponding confidence intervals (CIs). The Bonferroni correction was applied to adjust the P value. Specifically, the significance threshold was set at the number of 0.05/number of tests.

For the identified plasma proteins, we used their trans-pQTLs (>1 Mb from the transcription start site of the gene) as IVs and performed MR analyses with EM. Screening thresholds for IVs were P < 5x10^-8^, r^2^ < 0.1 and kb = 10000. The primary MR analyses method was IVW.

### Sensitivity analyses

#### SMR analyses and HEDI heterogeneity test

SMR was used as a complementary method to further validate causal associations between proteins and EM. This method allows the detection of pleiotropic associations between gene expression levels and complex traits of interest (21). The significance threshold for the SMR is set at P < 0.05/number of tests with Bonferroni correction in this study. HEIDI is a heterogeneity test that takes into account the linkage relationship between SNPs. The HEIDI test indicates that the causality between plasma proteins and EM is not confounded by linkage disequilibrium (LD) if the P value of the HEIDI test is >0.05.

#### Co-localization analyses

Co-localization analyses was used to identify whether circulating proteins and EM risk are influenced by a same causal variant, and to assess possible bias due to LD, thus providing the evidence for a correlation between the two phenotypes (22). The “COLOC” package was used to support the analyses of the data. Co-localization analyses within specific genomic regions assumes that there is at most one true causal variant per phenotype, involving a total of five mutually exclusive model assumptions (H0-H4). Namely, H0: No SNPs in this region are genetically associated with plasma proteins and EM risk; H1: SNPs in this region are only genetically associated with plasma proteins; H2: SNPs in this region are only genetically associated with endometriosis risk; H3: SNPs in this region are genetically associated with both proteins and EM risk, but using different causal variants; H4: SNPs in this region are genetically associated with both proteins and EM risk and share a causal variant. Each model yields a posterior probability (PPH0-PPH4), and the sum of the posterior probabilities of the five models is 1. The higher the posterior probability of a model, the more likely it is that the assumptions of that model are true given the data (23). In this study, we selected all SNPs in the 500 kb region upstream and downstream of the cis-pQTL for co-localization analyses. When PPH4>0.7, the genetic association between circulating protein levels and EM risk was supported by co-localization.

#### Reverse MR analyses and Steiger test

Reverse MR analyses was conducted with EM as the exposure and identified proteins as the outcome. SNPs with a P value less than 5×10^−8^ were selected as IVs. To ensure the independence between SNPs, the clump data function parameter (r^2^ = 0.001, kb=10000) was set to remove SNPs with LD. Sensitivity analyses refers to preliminary MR analyses. In addition, the hypothesis of the MR analyses is that IVs would first affect circulating protein levels and then influence the risk of EM through circulating protein levels. Thus, the directionality of this hypothesis needs to be tested. The Steiger analyses can separately calculate the variance explain of IVs on circulating protein levels and the risk of EM (24). If the variance explain of the risk of EM is less than the exposure, the direction is correct and the problem of endogeneity caused by reverse causality is avoided.

### Validated PWAS analyses

Functional summary-based imputation (FUSION) is an efficient algorithm that builds a predictive model of a functional/molecular phenotypic genetic component and uses GWAS summary statistics to predict and test the association of that component with EM. Five predictive models, including top1, blup, lasso, enet, and bslmm, were used for the FUSION analyses (25). To conduct the validated PWAS study of EM, we used the FUSION method to combine the genetic effect and protein weights of EM by calculating the linear product of Z-scores and protein weights for independent SNPs at a locus. Data were analysed using the “FUSION” package (https://github.com/gusevlab/fusion_twas/archive/master.zi).

### Protein-protein interaction and druggability evaluation

The STRING database (https://string-db.org/) was used to construct PPI networks to validate potential interactions between the proteins identified in this MR study and known EM drug targets. Moreover, the DGIdb v.5.0.3 database (https://www.dgidb.org/) was used to search for interactions between the identified proteins and drugs to assess whether these proteins could serve as potential therapeutic targets (26).

### Single-cell transcriptomic analyses

Single-cell transcriptomic analyses were performed to validate the expression of potential target genes in EM patients. Raw data were downloaded from the GSE203191 and GSE179640 datasets on GEO (https://www.ncbi.nlm.nih.gov/geo/). The Seurat software package (version 5.0.0) was utilized for data processing. GSE203191 included menstrual effluent (ME) samples from 33 subjects, including confirmed EM patients (cases), controls, and symptomatic subjects (who experiencing chronic symptoms of endometriosis but have not received a formal diagnosis). GSE179640 included eutopic endometrium (EuE) samples from 9 EM patients and 3 controls.

Following established protocols, we performed a rigorous quality control of the scRNA-seq data. We targeted on the top 2000 highly variable genes (HVGs) in each sample, which were determined by variance stabilizing transformation (vst) and normalized for subsequent analyses. Genes were scaled using the `ScaleData` function, and dimensionality reduction was performed using the `RunPCA` function with 30 dimensions (dim = 30). Batch correction for the two datasets was achieved using the integrated ` IntegrateLayers` function and ‘rpca’ method in Seurat.

Cell clustering was performed using the `FindNeighbors` and `FindClusters` functions, resulting in the identification of 14 distinct cell clusters. Visualization of these clusters was achieved using the `RunUMAP` function, providing a clear illustration of cellular heterogeneity and distinct cell populations within the dataset.

## Results

### Preliminary MR analyses

**Figure 1:**
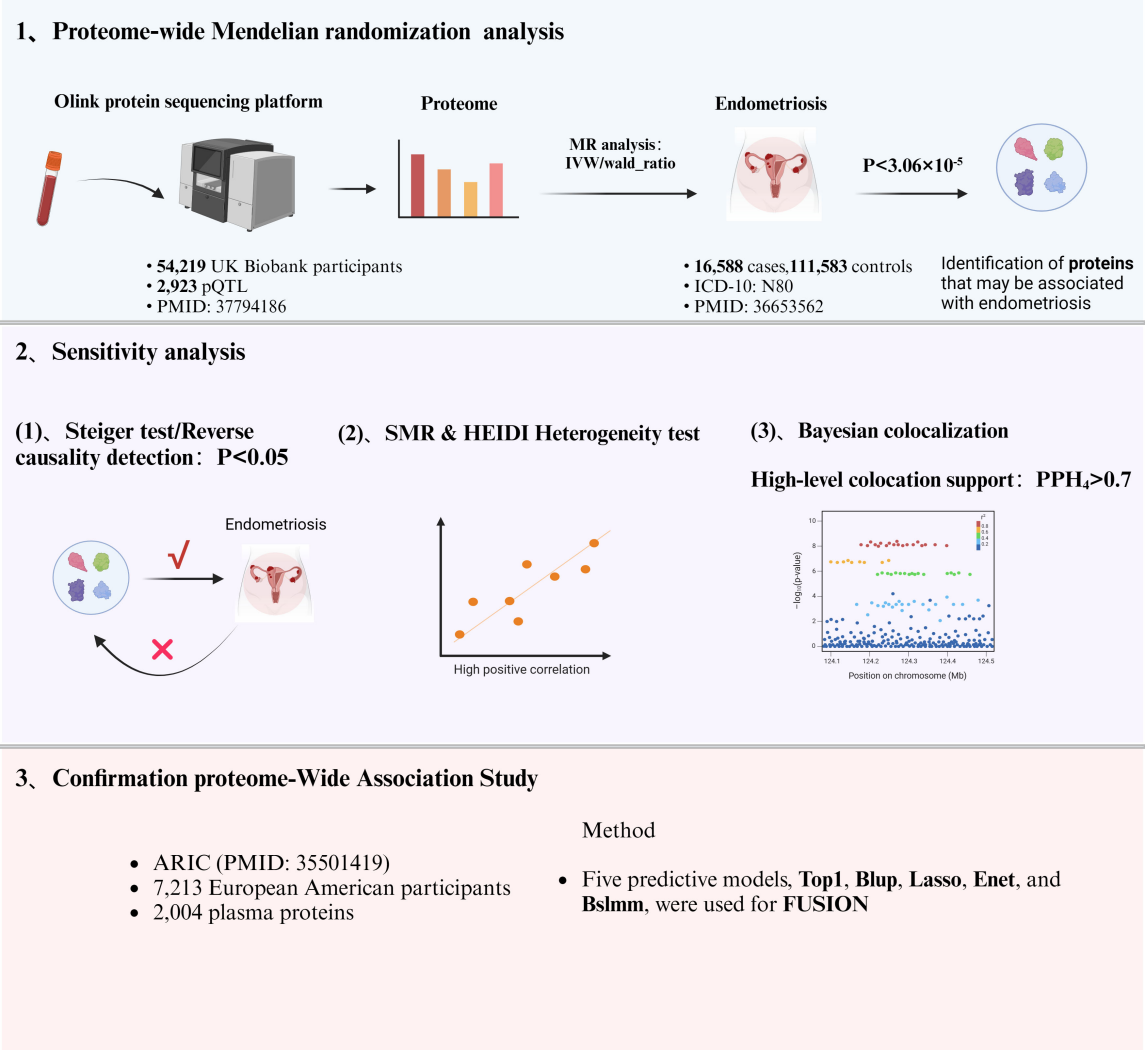
Flowchart of the research design.

**Figure 2:**
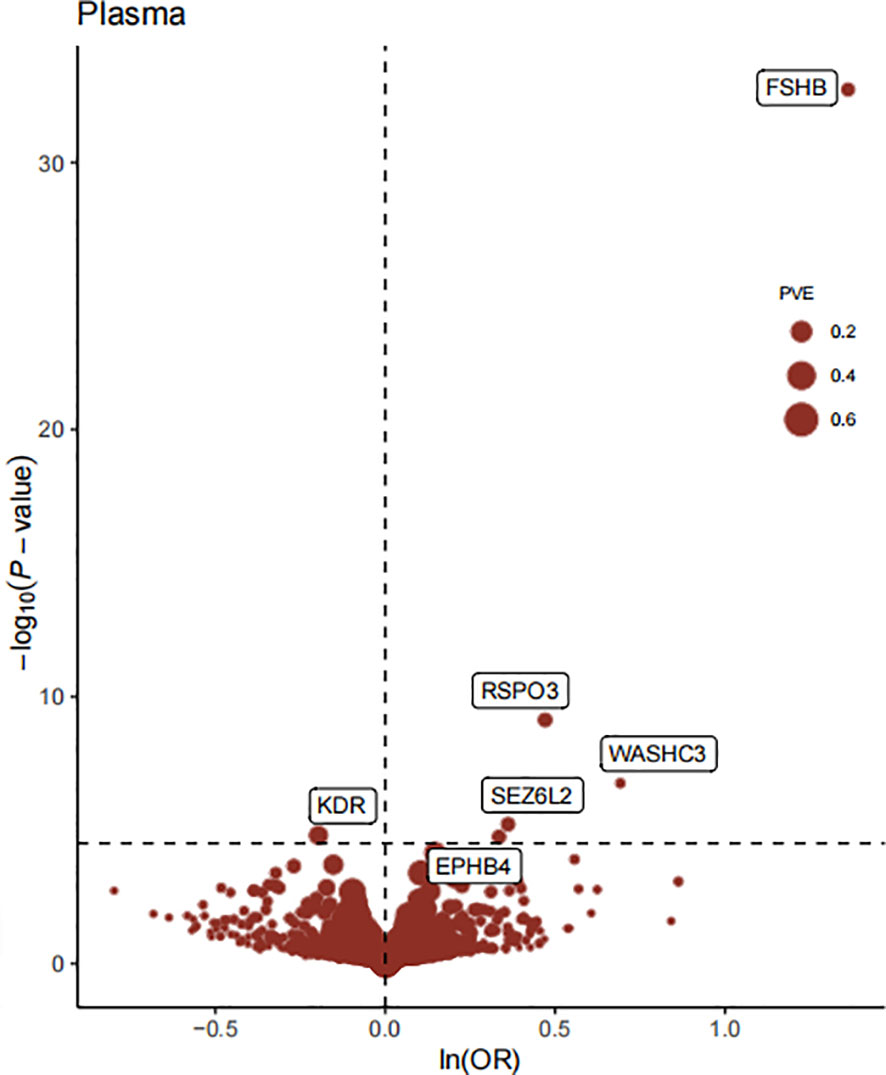
The Volcano plot of preliminary MR analyses results. The x-axis represents the OR value, the y-axis represents the -log10(P) value of the MR result, and the horizontal dashed line represents the corrected threshold of 3.06×10^-5^, with the size of the dot representing the PVE (percent variance explained).

The flowchart of the study design is shown in **Figure 1**. In this study, 1632 plasma proteins were investigated for causal association with EM (**Supplementary Table S1**). After Bonferroni adjustment of the hypothesis testing threshold (P<3.06×10^-5^, i.e, P<0.05/1632), as shown in **Figure 2**, the preliminary MR study identified six plasma proteins associated with the risk of EM, of which five plasma proteins were risk factors for EM, including ephrin type-B receptor 4 (EPHB4) (OR = 1.40, 95% CI: 1.20 - 1.63, P = 1.76×10^-5^), follitropin subunit beta (FSHB) (OR = 3.91, 95% CI: 3.13 - 4.87, P = 1.84×10^-33^), r-spondin-3 (RSPO3) (OR = 1.60, 95% CI: 1.38 - 1.86, P = 7.42×10^-10^), seizure 6-like protein 2 (SEZ6L2) (OR = 1.44, 95% CI: 1.23 - 1.68, P = 5.99×10^-6^), and WASH complex subunit 3 (WASHC3) (OR = 2.00, 95% CI: 1.54 - 2.59, P = 1.74×10^-7^). Additionally, vascular endothelial growth factor receptor 2 (KDR) was identified as a protective factor for EM (OR = 0.80, 95% CI: 0.75 - 0.90, P = 1.54×10^-5^). All results of the preliminary MR analyses are shown in **Supplementary Table S2**.

### Sensitivity analyses

To further validate the causal association identified by the MR analyses, we performed follow-up SMR and HEIDI heterogeneity tests on six plasma proteins (**Supplementary Table S3**, **Table 1**), which showed that all six plasma proteins passed the SMR test (P < 8.33 × 10^-3^, i.e, P<0.05/6), but only four plasma proteins passed the HEIDI heterogeneity test (PHEIDI > 0.05), including FSHB (PHEIDI = 0.112), RSPO3 (PHEIDI = 0.170), SEZ6L2 (PHEIDI = 0.189) and EPHB4 (PHEIDI = 0.682).

**Table 1:** Reverse MR analyses, Steiger filtering test, Bayesian co-localization and SMR results of the four identified plasma proteins.

Protein	Bidirectional MR (P_IVW_)	Steiger filtering	Co-localization PPH4	SMR (pval)	HEIDI (pval)
EPHB4	0.338	TRUE (7.97×10^-64^)	0.926	2.58×10^-5^	0.682
FSHB	0.816	TRUE (1.87×10^-35^)	0.998	3.99×10^-25^	0.112
RSPO3	0.096	TRUE (1.45×10^-65^)	0.740	2.89×10^-9^	0.170
SEZ6L2	0.257	TRUE (1.29×10^-67^)	0.974	9.28×10^-6^	0.189

Bidirectional MR, bidirectional Mendelian randomization; SMR, Summary-databased MR analyses; HEIDI, heterogeneity in dependent instruments; pval, P value; EPHB4, Ephrin type-B receptor 4; FSHB, Follitropin subunit beta; RSPO3, R-spondin-3; SEZ6L2, Seizure 6-like protein 2.

**Figure 3:**
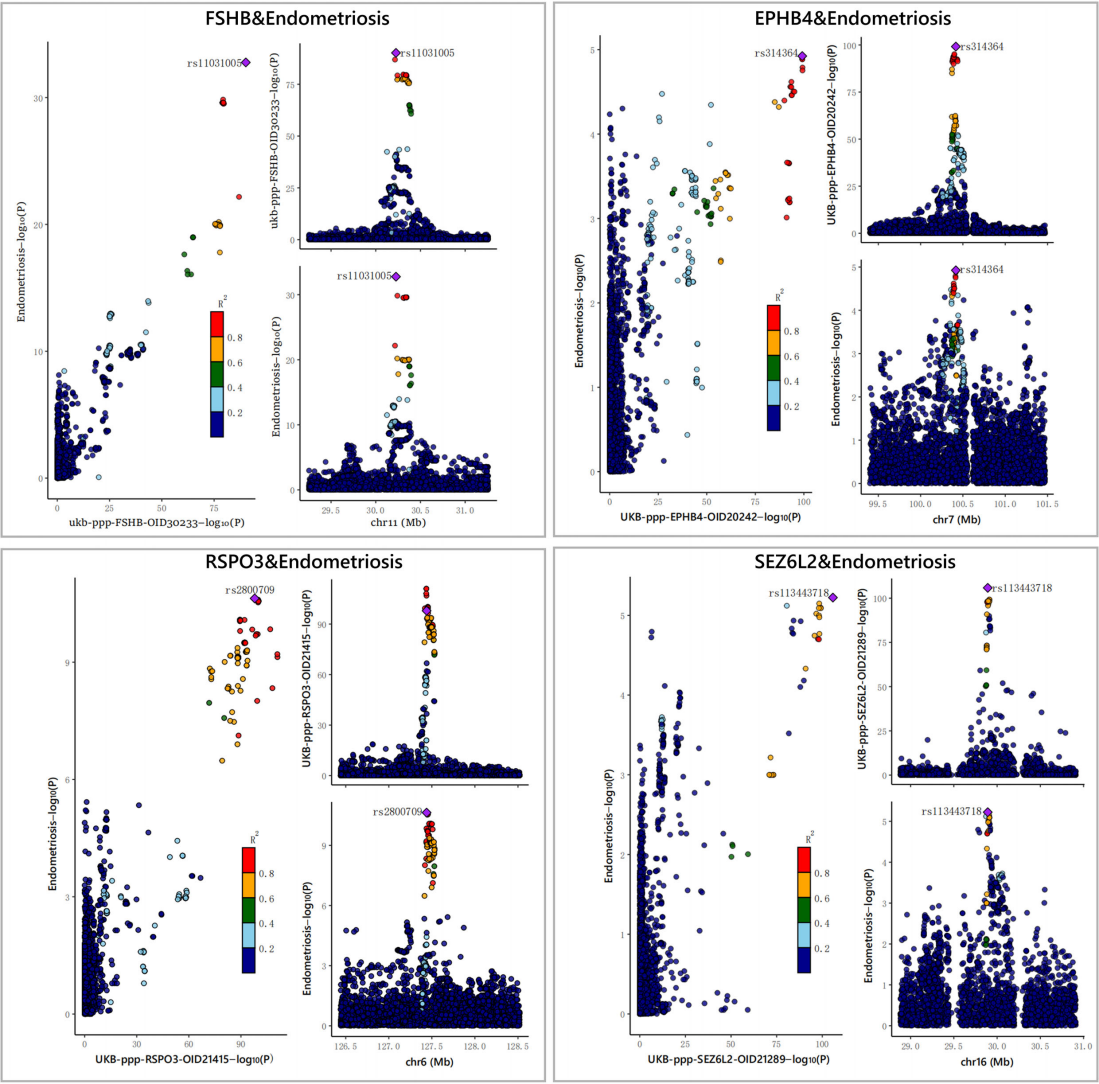
The results of the Bayesian co-localization analyses. The Bayesian co-localization analyses shows that FSHB, RSPO3, SEZ6L2 and EPHB4 received high support for co-localization with EM (PPH4>0.7).

Furthermore, to assess the possible confounding effect of the LD, we performed co-localization analyses of these four plasma proteins (**Supplementary Table S4**, **Table 1**). The result showed that all of them received high co-localization support (PPH4 > 0.7) (**Figure 3**).

After MR analyses, co-localization and SMR testing, we identified four plasma proteins as potential drug targets, including EPHB4, FSHB, RSPO3 and SEZ6L2. Reverse MR studies did not indicate a causal connection between EM and these four proteins (**Supplementary Tables S5**–**S7**). Steiger filtering further ensured the direction of the causal effect (**Table 1**). Regarding trans-pQTLs, our results suggest that trans-pQTLs of FSHB, RSPO3 and SEZ6L2 may be positively associated with EM risk, whereas trans-pQTLs of EPHB4 may have a negative association with EM risk (**Supplementary Tables S8**–**S10**).

### Validated PWAS analyses

**Figure 4:**
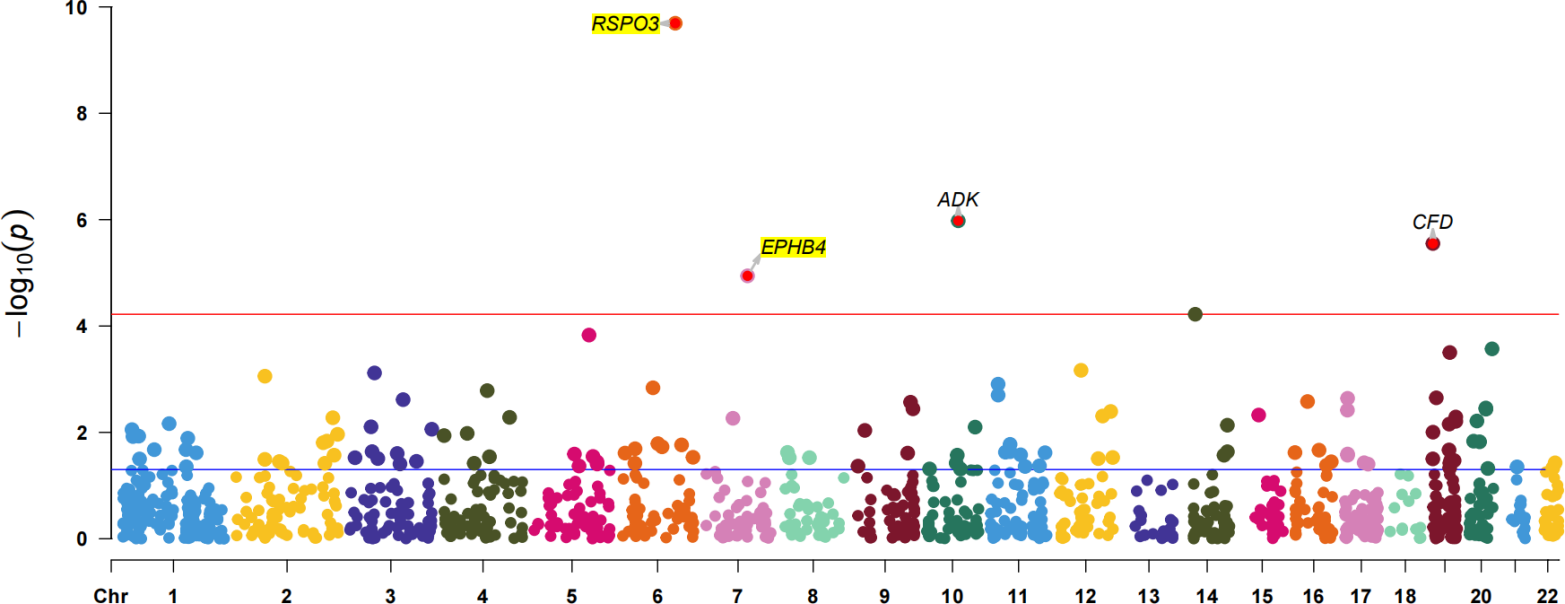
The Manhattan plot for validated PWAS study. The Manhattan plot shows the proteins identified by PWAS for EM. The red line indicates the Bonferroni significance threshold (P<3.71×10^-5^). The purple dashed line indicates the nominal significance level (P<0.05).

To further confirm the causal association of circulating proteins with EM, we also performed a validated PWAS analyses (**Supplementary Table S11**). After correcting for multiple hypothesis testing results using the Bonferroni method (P<3.71×10^-5^, i.e, P<0.05/1347), four plasma proteins were identified as causally associated with EM, including RSPO3 (Z = 6.36, PWAS.P = 2.03×10^-10^), ADK (Adenylate Kinase) (Z = -4.88, PWAS.P = 1.05×10^-6^), CFD (Complement Factor D) (Z = -4.68, PWAS.P = 2.81×10^-6^) and EPHB4 (Z = 4.39, PWAS.P = 1.14×10^-5^) (**Figure 4**). Among these, two plasma proteins, RSPO3 and EPHB4, confirmed the results of the preliminary MR study.

### PPI and druggability evaluation

Due to the limited number of proteins that have been identified, PPI shows that only a low scoring relationship between FSHB and SEZ6L2 (score 0.135), whose putative homologs are co-expressed in other organisms. In the druggability evaluation, we found that FSHB belongs to the hypothalamic-pituitary-gonadal axis, and only targeted drugs that block the gonadotropin releasing hormone (GnRH) receptor located upstream of the FSHB are available. It is worth noting that a number of other drugs developed to target EPHB4 have been shown to inhibit tumour angiogenesis, including VANDETANIB, which has received drug marketing approval. In addition, Rosmantuzumab (OMP-131R10) is an anti-RSPO3 monoclonal antibody currently in clinical trials for the treatment of advanced relapsed and refractory solid tumours.

### Single-cell transcriptomic analyses

**Figure 5:**
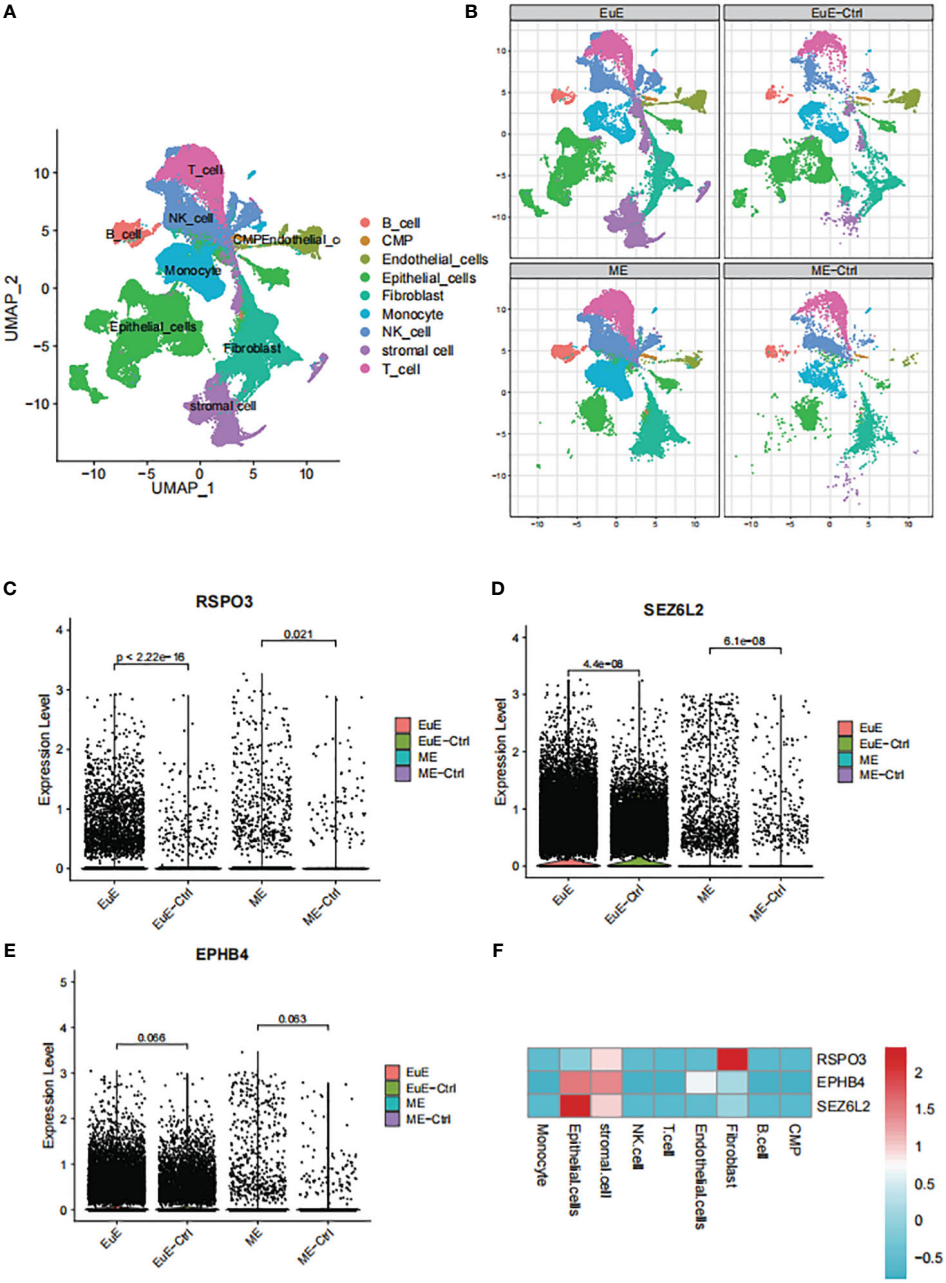
The single-cell transcriptomic analysis of EuE and ME samples from EM patients. **(A)** UMAP plot showing the distribution of 9 distinct cell populations. **(B)** Split UMAP plots showing the unique population of stromal cells and epithelial cells in the EuE samples from EM patients. **(C–E)** AUCell scoring and expression of RSPO3, SEZ6L2 and EPHB4 in EuE and ME samples from EM patients. **(F)** Heatmap showing the expression levels of RSPO3, SEZ6L2 and EPHB4 genes in 9 distinct cell populations.

Single-cell transcriptomic analyses of EuE and ME samples from EM patients and healthy controls revealed the presence of nine distinct immune cell populations (**Figure 5A**). Notably, a unique population of stromal cells was observed in the EuE samples of EM patients (**Figure 5B**). Additionally, significant differences were found in the epithelial cells of EuE samples of EM patients.

Upon analysis of the single-cell data, it was observed that FSHB was not expressed in either the EuE or ME samples. The lack of expression may be due to the fact that FSHB is a hormone secreted by the pituitary gland, which acts on the endometrium through hormonal regulation, rather than being expressed within the endometrium. However, RSPO3, SEZ6L2, and EPHB4 were observed to be expressed at higher levels in EM patients compared to controls in both EuE and ME samples (**Figures 5C–E**). Although the expression of EPHB4 was not statistically significant (p > 0.05), the trend was consistent. Furthermore, the expression levels of these genes were higher in EuE samples before being reflected in ME, indicating their initial role in the endometrial tissue. Our detailed analysis revealed that RSPO3 was predominantly expressed in stromal cells and fibroblasts. SEZ6L2 and EPHB4 were highly expressed in both stromal and epithelial cells (**Figure 5F**).

## Discussion

Currently, there are no treatments available that fully meet the clinical needs for EM management (5, 27). In this study, we investigated the causality between the plasma proteome and EM using MR analyses combined with SMR testing and co-localization analyses. PWAS and single-cell transcriptomic analyses were further performed to validated the findings. Our study revealed that genetically determined circulating levels of FSHB, RSPO3, SEZ6L2 and EPHB4 are causally associated with EM risk. These four proteins may be potential drug targets for EM. Furthermore, single-cell analyses have identified unique populations of stromal and epithelial cells in EuE samples from EM patients compared to controls. Notably, highly expression of SEZ6L2 and EPHB4 were observed in stromal and epithelial cells within EM lesions, while RSPO3 exhibited increased expression in stromal cells and fibroblasts.

FSHB is a hormone secreted by the pituitary gland that plays a crucial role in ovarian folliculogenesis and hormonal regulation (28). Recent studies have shown that a high-risk allele (rs74485684) located upstream of the FSHB promoter has high LD with SNPs related to FSH levels (rs11031005) and luteinizing hormone (LH) levels (rs11031002) in the EM population. Together, these SNPs are involved in regulating the release of FSH and LH, which affect the level of estrogen that plays a key role in EM (29). Our single-cell analyses found that FSHB was not expressed in either EuE or ME samples from EM patients, further supporting the previous view that it may be involved in the pathogenesis of EM by regulating hormone levels rather than being expressed within the endometrium. GnRH agonists and antagonists are currently the main drugs used to relieve EM symptoms and prevent recurrence. They mainly downregulate GnRH receptors in the hypothalamus and desensitise the pituitary gland. This causes the pituitary gland to produce less FSH and LH, which in turn reduces estrogen production in the ovaries. However, GnRH agonists and antagonists have a number of unavoidable side effects, including hot flushes, sweating, mood changes, osteoporosis and cardiovascular risk (30, 31). In this study, FSHB has been identified as a potential drug target for EM using a comprehensive genetic analyses approach including MR, SMR and co-localization analyses. This suggests that in the future, drugs could be developed to treat EM by targeting FSH release rather than blocking the entire hypothalamic-pituitary-ovarian axis.

Among the potential drug targets identified, EPHB4 is a receptor tyrosine kinase that plays a central role in angiogenesis, vascular remodelling and permeability (32, 33). Angiogenesis is an essential step in the development of the EM, and the vascular endothelial growth factor (VEGF) family and hypoxia inducible factor-1α(HIF-1α) play important roles in angiogenesis in endometriotic foci (34, 35). EPHB4 has been shown to interact with VEGF in several diseases and plays a key role in angiogenesis (36). In addition, EPHB4 can induce the expression of HIF-1α downstream genes by activating STAT3 (37). Our study showed that EPHB4 is highly expressed in both stromal and epithelial cells in EM lesions. Notably, previous studies have reported that inhibition of EPHB4 expression leads to a significant decrease in the number of proliferating stromal cells and a slower growth of ectopic lesions (38), suggesting that EPHB4 may be a promising therapeutic target for EM. There are currently some drugs that target EPHB4, such as VANDETANIB, which is approved to inhibit tumour angiogenesis and tumour cellproliferation. These drugs may also have potential in the treatment of EM.

RSPO3 is a member of the R-spondin protein family, which is usually associated with activation of the classical Wnt/β-catenin signalling pathway (39–41). In particular, the Wnt/β-catenin signalling pathway is closely associated with endometrial stromal cell proliferation, migration, invasion and fibrosis (42, 43). In this study, we found that RSPO3 was predominantly highly expressed in stromal cells and fibroblasts of EM lesions, suggesting a possible correlation between the pathogenesis of EM and the RSPO3-Wnt/β-catenin signaling pathway in stromal cells and fibroblasts. RSPO3 may be a potential drug target for the treatment of EM. Rosmantuzumab (OMP-131R10) is a monoclonal antibody that targets RSPO3 to attenuate classical WNT signalling. Rosmantuzumab is currently in clinical trials for the treatment of advanced relapsed and refractory solid tumours (44, 45). Its potential as a targeted agent for the treatment of EM warrants further investigation. SEZ6L2 is a seizure-associated cell surface protein now known to be primarily associated with autoimmune encephalitis and cerebellar ataxia (46, 47). So far, there has been limited research on SEZ6L2 and EM.

Currently, the gold standard for diagnosing EM is laparoscopic pathological biopsy, and many deep pelvic EM cannot be diagnosed early by blood tests and B-ultrasound scans (7). There is an urgent need to discover novel diagnostic biomarkers for EM. Through plasma proteomic studies, we identified the four plasma proteins, FSHB, RSPO3, SEZ6L2, and EPHB4, as possible biomarkers for EM. Furthermore, increased expression levels of RSPO3, SEZ6L2, and EPHB4 were observed in EuE and ME samples from individuals with EM, especially RSPO3 and SEZ6L2, suggesting that the detection of RSPO3 and SEZ6L2 in ME samples may have diagnostic potential for EM.

Our study has several limitations. First, our study concentrated mainly on European persons. Caution should be exercised in extrapolating these findings to other ethnic groups. Second, the result showed that the cis-pQTLs of EPHB4 were positively correlated with the risk of EM, whereas the trans-pQTLs of EPHB4 had the opposite effect on EM risk. Using single-cell analysis, we confirmed a significant upregulation of EPHB4 expression in stromal and epithelial cells of EuE lesions. Therefore, we speculate that the cis-pQTLs of EPHB4 may play the major role in disease risk, but the specific biological mechanisms remain to be further explored. In addition, for the other three potential drug targets, although the PWAS and single-cell analyses validated the findings, further mechanistic studies in EM patients will be required in the future.

## Conclusion

Our study identified FSHB, RSPO3, SEZ6L2, and EPHB4 as potential drug targets for EM and highlighted the critical role of stromal and epithelial cells in disease development. These findings provide new insights into the diagnosis and treatment of EM.

## Data availability statement

The original contributions presented in the study are included in the article/**Supplementary Material**. Further inquiries can be directed to the corresponding authors.

## Author contributions

TT: Conceptualization, Data curation, Formal Analysis, Funding acquisition, Software, Supervision, Writing – original draft, Writing – review & editing. XM: Conceptualization, Data curation, Formal Analysis, Supervision, Writing – original draft, Writing – review & editing. LZ: Conceptualization, Data curation, Formal Analysis, Funding acquisition, Software, Supervision, Writing – original draft, Writing – review & editing.

## References

[1] Saunders PTK Horne AW. Endometriosis: Etiology, pathobiology, and therapeutic prospects. Cell. (2021) 184:2807–24. doi: 10.1016/j.cell.2021.04.041 34048704

[2] Shafrir AL Farland LV Shah DK Harris HR Kvaskoff M Zondervan K. Risk for and consequences of endometriosis: A critical epidemiologic review. Best Pract Res Clin Obstet Gynaecol. (2018) 51:1–15. doi: 10.1016/j.bpobgyn.2018.06.001 30017581

[3] Soliman AM Surrey E Bonafede M Nelson JK Castelli-Haley J. Real-world evaluation of direct and indirect economic burden among endometriosis patients in the United States. Adv Ther. (2018) 35:408–23. doi: 10.1007/s12325-018-0667-3 PMC5859693 29450864

[4] Taylor HS Kotlyar AM Flores VA. Endometriosis is a chronic systemic disease: clinical challenges and novel innovations. Lancet. (2021) 397:839–52. doi: 10.1016/S0140-6736(21)00389-5 33640070

[5] Arafah M Rashid S Akhtar M. Endometriosis: A comprehensive review. Adv Anat Pathol. (2021) 28:30–43. doi: 10.1097/PAP.0000000000000288 33044230

[6] Giudice LC Kao LC. Endometriosis. Lancet. (2004) 364:1789–99. doi: 10.1016/S0140-6736(04)17403-5 15541453

[7] Koninckx PR Fernandes R Ussia A Schindler L Wattiez A Al-Suwaidi S. Pathogenesis based diagnosis and treatment of endometriosis. Front Endocrinol (Lausanne). (2021) 12:745548. doi: 10.3389/fendo.2021.745548 34899597 PMC8656967

[8] Ochoa D Karim M Ghoussaini M Hulcoop DG McDonagh EM Dunham I. Human genetics evidence supports two-thirds of the 2021 FDA-approved drugs. Nat Rev Drug Discovery. (2022) 21:551. doi: 10.1038/d41573-022-00120-3 35804044

[9] Zheng J Haberland V Baird D Walker V Haycock PC Hurle MR. Phenome-wide Mendelian randomization mapping the influence of the plasma proteome on complex diseases. Nat Genet. (2020) 52:1122–31. doi: 10.1038/s41588-020-0682-6 PMC7610464 32895551

[10] Sun BB Chiou J Traylor M Benner C Hsu YH Richardson TG. Plasma proteomic associations with genetics and health in the UK Biobank. Nature. (2023) 622:329–38. doi: 10.1038/s41586-023-06592-6 PMC10567551 37794186

[11] Moncla LM Mathieu S Sylla MS Bossé Y Thériault S Arsenault BJ. Mendelian randomization of circulating proteome identifies actionable targets in heart failure. BMC Genomics. (2022) 23:588. doi: 10.1186/s12864-022-08811-2 35964012 PMC9375407

[12] Lin J Zhou J Xu Y. Potential drug targets for multiple sclerosis identified through Mendelian randomization analysis. Brain. (2023) 146:3364–72. doi: 10.1093/brain/awad070 PMC10393411 36864689

[13] Wu Y Wang Z Yang Y Han C Wang L Kang K. Exploration of potential novel drug targets and biomarkers for small cell lung cancer by plasma proteome screening. Front Pharmacol. (2023) 14:1266782. doi: 10.3389/fphar.2023.1266782 37745050 PMC10511877

[14] Wu BS Chen SF Huang SY Ou YN Deng YT Chen SD. Identifying causal genes for stroke via integrating the proteome and transcriptome from brain and blood. J Transl Med. (2022) 20:181. doi: 10.1186/s12967-022-03377-9 35449099 PMC9022281

[15] Zhang J Dutta D Köttgen A Tin A Schlosser P Grams ME. Plasma proteome analyses in individuals of European and African ancestry identify cis-pQTLs and models for proteome-wide association studies. Nat Genet. (2022) 54:593–602. doi: 10.1038/s41588-022-01051-w 35501419 PMC9236177

[16] Kurki MI Karjalainen J Palta P Sipilä TP Kristiansson K Donner KM. FinnGen provides genetic insights from a well-phenotyped isolated population. Nature. (2023) 613:508–18. doi: 10.1038/s41586-022-05473-8 PMC9849126 36653562

[17] Zhu Z Zhang F Hu H Bakshi A Robinson MR Powell JE. Integration of summary data from GWAS and eQTL studies predicts complex trait gene targets. Nat Genet. (2016) 48:481–7. doi: 10.1038/ng.3538 27019110

[18] Sanderson E Glymour MM Holmes MV Kang H Morrison J Munafò MR. Mendelian randomization. Nat Rev Methods Primers. (2022). doi: 10.1038/s43586-021-00092-5 PMC7614635 37325194

[19] Hemani G Zheng J Elsworth B Wade KH Haberland V Baird D. The MR-Base platform supports systematic causal inference across the human phenome. Elife. (2018) 7. doi: 10.7554/eLife.34408 PMC5976434 29846171

[20] Burgess S Butterworth A Thompson SG. Mendelian randomization analysis with multiple genetic variants using summarized data. Genet Epidemiol. (2013) 37:658–65. doi: 10.1002/gepi.21758 PMC4377079 24114802

[21] Verbanck M Chen CY Neale B Do R. Publisher Correction: Detection of widespread horizontal pleiotropy in causal relationships inferred from Mendelian randomization between complex traits and diseases. Nat Genet. (2018) 50:1196. doi: 10.1038/s41588-018-0164-2 29967445

[22] Giambartolomei C Vukcevic D SChadt EE Franke L Hingorani AD Wallace C. Bayesian test for colocalisation between pairs of genetic association studies using summary statistics. PloS Genet. (2014) 10:e1004383. doi: 10.1371/journal.pgen.1004383 24830394 PMC4022491

[23] Foley CN Staley JR Breen PG Sun BB Kirk PDW Burgess S. A fast and efficient colocalization algorithm for identifying shared genetic risk factors across multiple traits. Nat Commun. (2021) 12:764. doi: 10.1038/s41467-020-20885-8 33536417 PMC7858636

[24] Hemani G Tilling K Davey Smith G. Orienting the causal relationship between imprecisely measured traits using GWAS summary data. PloS Genet. (2017) 13:e1007081. doi: 10.1371/journal.pgen.1007081 29149188 PMC5711033

[25] Gusev A Ko A Shi H Bhatia G Chung W Penninx BW. Integrative approaches for large-scale transcriptome-wide association studies. Nat Genet. (2016) 48:245–52. doi: 10.1038/ng.3506 PMC4767558 26854917

[26] Freshour SL Kiwala S Cotto KC Coffman AC McMichael JF Song JJ. Integration of the Drug-Gene Interaction Database (DGIdb 4.0) with open crowdsource efforts. Nucleic Acids Res. (2021) 49:D1144–d51. doi: 10.1093/nar/gkaa1084 PMC7778926 33237278

[27] Edi R Cheng T. Endometriosis: evaluation and treatment. Am Fam Physician. (2022) 106:397–404. 36260896

[28] Kumar TR. Rerouting of follicle-stimulating hormone secretion and gonadal function. Fertil Steril. (2023) 119:180–3. doi: 10.1016/j.fertnstert.2022.12.005 PMC10014147 36496082

[29] Sapkota Y Steinthorsdottir V Morris AP Fassbender A Rahmioglu N De Vivo I. Meta-analysis identifies five novel loci associated with endometriosis highlighting key genes involved in hormone metabolism. Nat Commun. (2017) 8:15539. doi: 10.1038/ncomms15539 28537267 PMC5458088

[30] Capezzuoli T Rossi M La Torre F Vannuccini S Petraglia F. Hormonal drugs for the treatment of endometriosis. Curr Opin Pharmacol. (2022) 67:102311. doi: 10.1016/j.coph.2022.102311 36279764

[31] Veth VB van de Kar MM Duffy JM van Wely M Mijatovic V Maas JW. Gonadotropin-releasing hormone analogues for endometriosis. Cochrane Database Syst Rev. (2023) 6:Cd014788. doi: 10.1002/14651858.CD014788.pub2 37341141 PMC10283345

[32] Salvucci O Tosato G. Essential roles of EphB receptors and EphrinB ligands in endothelial cell function and angiogenesis. Adv Cancer Res. (2012) 114:21–57. doi: 10.1016/B978-0-12-386503-8.00002-8 22588055 PMC3500853

[33] Zeng X Hunt A Jin SC Duran D Gaillard J Kahle KT. EphrinB2-ephB4-RASA1 signaling in human cerebrovascular development and disease. Trends Mol Med. (2019) 25:265–86. doi: 10.1016/j.molmed.2019.01.009 PMC6456402 30819650

[34] Zhang M Xu T Tong D Li S Yu X Liu B. Research advances in endometriosis-related signaling pathways: A review. BioMed Pharmacother. (2023) 164:114909. doi: 10.1016/j.biopha.2023.114909 37210898

[35] Powell SG Sharma P Masterson S Wyatt J Arshad I Ahmed S. Vascularisation in deep endometriosis: A systematic review with narrative outcomes. Cells. (2023) 12. doi: 10.3390/cells12091318 PMC10177118 37174718

[36] Martiny-Baron G Holzer P Billy E Schnell C Brueggen J Ferretti M. The small molecule specific EphB4 kinase inhibitor NVP-BHG712 inhibits VEGF driven angiogenesis. Angiogenesis. (2010) 13:259–67. doi: 10.1007/s10456-010-9183-z PMC2941628 20803239

[37] Su Q Wang J Wu Q Ullah A Ghauri MA Sarwar A. Sanguinarine combats hypoxia-induced activation of EphB4 and HIF-1α pathways in breast cancer. Phytomedicine. (2021) 84:153503. doi: 10.1016/j.phymed.2021.153503 33636580

[38] Rudzitis-Auth J Fuß SA Becker V Menger MD Laschke MW. Inhibition of erythropoietin-producing hepatoma receptor B4 (EphB4) signalling suppresses the vascularisation and growth of endometriotic lesions. Br J Pharmacol. (2020) 177:3225–39. doi: 10.1111/bph.15044 PMC7312277 32144768

[39] Madan B Virshup DM. Targeting Wnts at the source–new mechanisms, new biomarkers, new drugs. Mol Cancer Ther. (2015) 14:1087–94. doi: 10.1158/1535-7163.MCT-14-1038 25901018

[40] Kazanskaya O Ohkawara B Heroult M Wu W Maltry N Augustin HG. The Wnt signaling regulator R-spondin 3 promotes angioblast and vascular development. Development. (2008) 135:3655–64. doi: 10.1242/dev.027284 18842812

[41] Sharma AR Chakraborty C Lee SS Sharma G Yoon JK George Priya Doss C. Computational biophysical, biochemical, and evolutionary signature of human R-spondin family proteins, the member of canonical Wnt/β-catenin signaling pathway. BioMed Res Int. (2014) 2014:974316. doi: 10.1155/2014/974316 25276837 PMC4172882

[42] Matsuzaki S Darcha C. Involvement of the Wnt/β-catenin signaling pathway in the cellular and molecular mechanisms of fibrosis in endometriosis. PloS One. (2013) 8:e76808. doi: 10.1371/journal.pone.0076808 24124596 PMC3790725

[43] Matsuzaki S Botchorishvili R Pouly JL Canis M. Targeting the Wnt/β-catenin pathway in endometriosis: a potentially effective approach for treatment and prevention. Mol Cell Ther. (2014) 2:36. doi: 10.1186/s40591-014-0036-9 26056600 PMC4451963

[44] Zhang M Haughey M Wang NY Blease K Kapoun AM Couto S. Targeting the Wnt signaling pathway through R-spondin 3 identifies an anti-fibrosis treatment strategy for multiple organs. PloS One. (2020) 15:e0229445. doi: 10.1371/journal.pone.0229445 32160239 PMC7065809

[45] Katoh M. Multi−layered prevention and treatment of chronic inflammation, organ fibrosis and cancer associated with canonical WNT/β−catenin signaling activation (Review). Int J Mol Med. (2018) 42:713–25. doi: 10.3892/ijmm.2018.3689 PMC6034925 29786110

[46] Landa J Guasp M Petit-Pedrol M Martínez-Hernández E Planagumà J Saiz A. Seizure-related 6 homolog like 2 autoimmunity: Neurologic syndrome and antibody effects. Neurol Neuroimmunol Neuroinflamm. (2021) 8. doi: 10.1212/NXI.0000000000000916 PMC7641326 33144342

[47] Abe M Yaguchi H Kudo A Nagai A Shirai S Takahashi-Iwata I. Sez6l2 autoimmunity in a large cohort study. J Neurol Neurosurg Psychiatry. (2023) 94:667–8. doi: 10.1136/jnnp-2022-330194 37263766

